# Improvisational Movement to Improve Quality of Life in Older Adults With Early-Stage Dementia: A Pilot Study

**DOI:** 10.3389/fspor.2021.796101

**Published:** 2022-01-14

**Authors:** Deepthi Thumuluri, Robert Lyday, Phyllis Babcock, Edward H. Ip, Robert A. Kraft, Paul J. Laurienti, Rebecca Barnstaple, Christina T. Soriano, Christina E. Hugenschmidt

**Affiliations:** ^1^Section on Gerontology and Geriatric Medicine, Wake Forest School of Medicine, Winston-Salem, NC, United States; ^2^Department of Radiology, Wake Forest School of Medicine, Winston-Salem, NC, United States; ^3^Department of Biostatistics and Data Science, Wake Forest School of Medicine, Winston-Salem, NC, United States; ^4^Translational Science Center, Wake Forest University, Winston-Salem, NC, United States; ^5^Departments of Dance and Psychology, York University, Toronto, ON, Canada; ^6^Department of Theatre and Dance, Wake Forest University, Winston-Salem, NC, United States

**Keywords:** Alzheimer's disease, dance, aging, brain, caregiver, older adult

## Abstract

Alzheimer's disease has profound effects on quality of life, affecting not only cognition, but mobility and opportunities for social engagement. Dance is a form of movement that may be uniquely suited to help maintain quality of life for older adults, including those with dementia, because it inherently incorporates movement, social engagement, and cognitive stimulation. Here, we describe the methods and results of the pilot study for the IMOVE trial (NCT03333837, www.clinicaltrials.gov), a clinical trial designed to use improvisational dance classes to test the effects of movement and social engagement in people with mild cognitive impairment (MCI) or early-stage dementia. The pilot study was an 8-week investigation into the feasibility and potential effects of an improvisational dance intervention on people with MCI or early-stage dementia (PWD/MCI) and their caregivers (CG). The pilot aimed to assess changes in quality of life, balance, mood, and functional brain networks in PWD/MCI and their CG. Participants were recruited as dyads (pairs) that included one PWD/MCI and one CG. Ten total dyads were enrolled in the pilot study with five dyads assigned to the usual care control group and five dyads participating in the dance intervention. The intervention arm met twice weekly for 60 min for 8 weeks. Attendance and quality of life assessed with the Quality of Life in Alzheimer's disease (QoL-AD) questionnaire were the primary outcomes. Secondary outcomes included balance, mood and brain network connectivity assessed through graph theory analysis of functional magnetic resonance imaging (fMRI). Class attendance was 96% and qualitative feedback reflected participants felt socially connected to the group. Increases in quality of life and balance were observed, but not mood. Brain imaging analysis showed increases in multiple brain network characteristics, including global efficiency and modularity. Further investigation into the positive effects of this dance intervention on both imaging and non-imaging metrics will be carried out on the full clinical trial data. Results from the trial are expected in the summer of 2022.

## Introduction

Alzheimer's disease (AD) is the most prevalent form of dementia, the most common neurodegenerative disease in older adults, and the 6th leading cause of death in the United States. In addition to cognitive impairments, people with AD and related dementias experience secondary symptoms that increase medical costs and decrease quality of life for the person with dementia (PWD) and their caregivers (CG), including neuropsychiatric symptoms such as apathy and depression, and motor symptoms such as altered gait and poor balance (Schubert et al., 2008; Gaugler et al., 2009; Okura and Langa, 2011). There is mounting evidence that sensory and motor regions of the central nervous system are affected by the pathology of AD (Albers et al., 2015); thus, interventions that target and support the sensorimotor system in combination with cognitive challenges may enhance patient function as AD progresses or delay the onset of more debilitating symptoms.

Changes in behavior and cognition in people with AD arise from neurodegeneration that has profound effects on brain function. Graph theory-based analysis of resting state functional MRI (fMRI) shows that brain networks in older adults with AD show increased path length, decreased global efficiency, and altered modular organization (Tijms et al., 2013; Dennis and Thompson, 2014). Furthermore, changes in functional connectivity in older adults with MCI are associated with gait (Hsu et al., 2019). Healthy older adults also show decreased global efficiency and modularity that are associated with mobility and cognition (Achard and Bullmore, 2007; Meunier et al., 2009; Hugenschmidt et al., 2014). Importantly, movement and improved fitness may improve overall brain health and plasticity (Voss et al., 2019), resulting in improved functional connectivity. Aerobic exercise interventions in older adults can increase brain volumes (Erickson et al., 2011), brain-derived neurotrophic factor (BDNF) (Erickson et al., 2011), and organization of functional networks (Burdette et al., 2010; Voss et al., 2010; Baniqued et al., 2017). Emerging evidence suggests that dance may also increase brain volumes (Rehfeld et al., 2018; Rektorova et al., 2020), structural connectivity (Burzynska et al., 2017), BDNF (Rehfeld et al., 2018), and functional connectivity (Zilidou et al., 2018; Balazova et al., 2021) in older adults.

Dance participation may be a powerful means of improving function and quality of life for PWD. A 2003 epidemiological study identified dance as the only leisure-time physical activity associated with reduced risk for dementia (Verghese et al., 2003). Since then, evidence has been accumulating through multiple smaller studies that the cognitively and socially stimulating movement that occurs in dance classes may improve cardiorespiratory fitness (Belardinelli et al., 2008; Rehfeld et al., 2018; Rodrigues-Krause et al., 2018), reduce depression (Eyigor et al., 2009), improve balance, and reduce fall risk in older adults (Eyigor et al., 2009; Kattenstroth et al., 2013). A number of recent systematic reviews and meta-analyses have demonstrated multimodal benefits of dance interventions in healthy older adults, including significant improvements in mobility and endurance (Liu et al., 2021) and contributions to the maintenance or improvement of aspects of cognition (Predovan et al., 2019). In healthy older adults, global cognitive functions and executive function (Hewston et al., 2021) have also been shown to be improved by dance, along with memory function (Meng et al., 2020). Most studies investigating dance in PWD have focused on cognitive outcomes. In older adults with mild cognitive impairment, dance has been seen to improve global cognition, attention, recall, and visuospatial ability (Chan et al., 2020). A two-armed quasi-experimental study by Dominguez et al. (2018) found that older adults with MCI improved on several cognitive measures, including the Alzheimer's Disease Assessment Scale-Cognitive (ADAS-Cog), Montreal Cognitive Assessment (MoCA-P), and the Boston Naming Test (BNT). In a recent RCT (Lazarou et al., 2017), Lazarou and colleagues found significant differences in performance on neuropsychological tests for elderly patients with amnestic MCI who participated in a ballroom dance intervention vs. controls. A recent systematic review and meta-analysis (Zhu et al., 2020) on the effects of aerobic dance on cognition in older adults with MCI found evidence for significant improvements in global cognitive function, memory, and executive function. However, as the authors report, more RCTs with improved designs and larger sample sizes are needed to clarify results.

While research into the benefits of dance for older adults has shown improvements across physical and cognitive domains, the multimodal and integrated nature of dance does not easily fit with the demands of traditional study designs such as blinding of researchers/participants, isolating variables, and limiting cross-factorial influences. Research capable of capturing the complexity of interactions while determining mechanisms and respecting the integrity of the form under investigation can be challenging to design and deliver. In addition, as this is a relatively young area of research, aspects such as effect size and dose for dance interventions are yet to be determined in many cases.

To address these challenges and increase our understanding of potential benefits of dance for people with Alzheimer's disease and their CG, we conducted a pilot study to assess feasibility and generate effect sizes for 8 weeks of improvisational dance classes compared to standard of care in people with MCI or early-stage dementia (PWD/MCI). The intervention used the IMPROVment® method, which is grounded in 4 principles of improvisation that shape the tone of the class: non-judgment, non-competitiveness, curiosity, and playfulness (Batson et al., 2016). This method was used in a previous study (Batson et al., 2014), which showed improved balance and mobility in people with Parkinson's disease, as well as markedly changed functional brain network architecture in the one individual scanned. Ongoing community classes as well as the largest support group for people with Parkinson's disease in Forsyth County, North Carolina, USA arose from this study, demonstrating that powerful social connections had formed in addition to visibly improved movement quality. Outcomes of interest included secondary symptoms of AD that are important for quality of life, including balance, gait, and neuropsychiatric symptoms, and changes in functional brain networks assessed with graph theory analysis of resting-state functional magnetic resonance imaging (fMRI) scans. We hypothesized that attendance would be >85% and participants in the dance intervention would show improvement or maintenance of quality of life, balance, and mood compared to those in the usual care control group. We further hypothesized that participants in the dance group would show increased connectivity in networks involved with movement and mood, specifically somatomotor regions and the default mode network. Results of this pilot study are reported here, but should not be interpreted as definitive outcomes given the extremely limited sample size and the pilot nature of the trial.

## Methods

### Overall Pilot Study Design

Participants were enrolled in the pilot study between 9/24/2015 and 10/23/2015 in Forsyth County, NC. After completing telephone screening and providing written, informed consent, participants completed one pre-intervention study visit that included a 30-min MRI scan along with assessments of gait, balance, and neuropsychiatric symptoms. At the end of the baseline visit, participants were informed of their assigned study group and scheduled for a follow-up visit. All intervention participants completed the follow-up study visit within 1 week of finishing the 8-week intervention. Participants in the control group were told to maintain their usual care and lifestyle. Maintenance was evaluated at the follow-up visits 8–9 weeks after by asking about any changes in medications, supplements, or health events. Because the protocol specified that the pre-intervention study visit was to be completed within 2 weeks of beginning the intervention, the first 5 dyads who completed baseline testing were assigned to the dance group. All participants were offered the dance intervention free of charge after completion of the pilot study through a community class. Due to the limited resources of the pilot trial, study staff were not able to be blinded. These study design issues are addressed in the full trial, where participants are randomized and study staff are blinded for all study visits.

### Pilot Study Participant Characteristics and Eligibility

A list of potential participants was provided by the Wake Forest Kulynych Memory Assessment Clinic (MAC) of people who had been given a diagnosis of MCI or early-stage AD within the past 6 months and lived within 45 min of the medical center. The pilot study contacted and assessed 18 dyads for participation. Of the 18, four dyads were excluded due to health issues and four elected to not participate. Ultimately, the study enrolled PWD (*n* = 10) aged 60–90 years and their CGs (*n* = 10) to participate in dyads (pairs) together in the study. CGs were required to have an active role in the study including participating in the intervention with the PWD. Potential PWD participants had a clinical or research diagnosis of MCI or early-stage AD (including mixed AD/vascular dementia) given through the Wake Forest Kulynych MAC or Wake Forest Alzheimer's Disease Research Center (ADRC). The MAC and ADRC use a highly overlapping battery of tests that includes a neuropsychological test battery, functional status questionnaires completed by a proxy, and a history and physical performed by a board-certified geriatrician or neurologist. The cognitive test battery consists of tests of naming, word fluency, working and episodic verbal and visual memory, and global cognition (in the ADRC, specifically the UDSv3). Depression and other psychiatric symptoms, functional independence, and CG assessment of cognitive and physical function are assessed with interviews and questionnaires. After reviewing all data and in consultation with the diagnostic team, dementia experts assign a diagnosis of AD, other forms of dementia, MCI, or normal cognition using Alzheimer's Association/NIA criteria (Weintraub et al., 2009). Potential participants were excluded for stroke, other causes of dementia, other neurological diseases, MRI incompatibility, or any major medical problem that could reasonably affect cognition or brain imaging measures, such as current cancer treatment. Results are reported on the PWD, as they were the primary target of the intervention.

### Intervention Description

#### The IMPROVment® Method

The intervention involved 1-h group improvisational dance sessions twice weekly for 8 weeks using the IMPROVment® method (improvment.wfu.edu) (Batson et al., 2014, 2016). Classes took place at a local dance studio (Academy of Dance Arts, Winston-Salem, NC, USA) located >0.5 miles from the medical center that is disability accessible. The room in which the classes took place had a mirrored wall, smooth dance surface, and wall-mounted barres. The classes were taught by Christina Soriano, MFA an associate professor of dance. She is a choreographer with extensive experience teaching modern dance, and the founder of the IMPROVment® method.

Classes were structured in four phases that included: (1) group warm-up in chairs positioned in a circle, (2) standing barre or back of the chair with solo and responsive exercises, (3) moving as a group through free space (with and without a partner), and (4) recuperation and rest. All exercises could be adapted for sitting if participants were unable for any reason to perform exercises at the barre or moving through free space. The choice to stand or walk was always self-selected by the participant. Similar exercises could appear within each phase (seated/standing/walking), creating a progression of motor skills, but the class series did not build incrementally. Recuperative phases are essential to rest and recover metabolically and cognitively. Recuperative movements were slower, simpler, and often more familiar (e.g., seated hamstring stretch). Active imagination, variability and pacing were primary components of each class that contributed to the overall tone of each class and support central principles of improvisation.

##### Active Imagination

Working with imagery is crucial in improvisatory practice and was an essential element of teaching in this pilot study. Verbal cues were used to create scenarios that cued or activated the imagination. In IMPROVment classes, verbal cueing takes primacy over entrainment to music, although the music itself may be used as an improvisatory cue. After calling out a cue, the teacher, who often acts more as a facilitator than an expert instructor, might demonstrate an optional response, but asks participants to respond with their own gestural inventions. As an example, students might be prompted during the seated warm-up to move in different imagined environments, such as water, a desert, or a windstorm. Verbal cues direct motor imagination by using rich language to encourage exploration of various movement qualities and inspire play.

##### Variability

Improvisational cues do not aim to teach specific patterns of response or habituate to them. Instead, they exist to self-generate possibilities for each individual based on their physical, emotional, and cognitive capabilities. Cues are delivered quickly, one after another. Within an average of 2 min after beginning class, tasks requiring quicker decision-making are introduced. Physical and cognitive challenges are advanced by dual- and multi-tasking. An example is a cue to direct traffic with the right side of the body while picking apples from an imaginary tree with the left.

##### Pacing

The pace of class references the rate at which new movement prompts are presented. Quick changes in pace avoid defaulting to habitual responses, thereby facilitating new options. Participants cannot rely on copying one another, memory, or anticipation to address challenges. For example, verbal cues are often delivered in a rapid-fire manner to prevent participants from reflecting on choices, changing their minds, or becoming embarrassed or dissatisfied with the choice made. The pace of class and the democratization of each cue helps also eliminate the hierarchy of the instructor as the “expert.”

Overall, these core tenants of class create a sense of joy, excitement and autonomy for participants and in a safe environment where risk taking is encouraged and celebrated.

#### Use of Music

Music is presented in a random fashion, as improvisational dance does not require entrainment of movement to music; musical atmosphere may be unrelated to movement instruction and merely ambient. Music can serve as improvisational cue, for example, participants may be invited to “play” an instrument they can hear in a complex piece of classical or jazz music. At times, participants themselves are invited to create music or sound accompaniment through vocalizing or body-based percussive actions. During this study, music was selected at random from the instructor's music library and included jazz, classical, big band/swing, and pop music from different eras spanning the 1950s to the present.

### Non-imaging Measures

Balance was assessed with the Fullerton Advanced Balance Scale (FAB), which measures balance using 10 different performance-based tests (Rose et al., 2006). A higher FAB score indicates better balance. Balance confidence was assessed using the Falls Efficacy Scale International (FES-I) (Yardley et al., 2005), a 16-item scale where higher scores reflect a higher risk of falling. Mood in both the PWD and CG was assessed using the Geriatric Depression Scale (Yesavage et al., 1982), a 15-item depression scale created for use in older adults. Quality of life in the PWD was assessed using the Quality of Life AD (QOL-AD) (Logsdon et al., 2002, 2007), a 13-item scale designed to assess quality of life in people with dementia. Neuropsychiatric symptoms were measured with the Neuropsychiatric Inventory (NPI) (Cummings, 1997), a structured interview with the CG that rates the frequency, severity, and distress of symptoms in the PWD across 12 domains. Given previous work suggesting that CG personality may influence CG reports of neuropsychiatric symptoms and burden (Orgeta and Leung, 2015), CG also completed the NEO Five Factor Inventory (NEO-FFI) (Costa and McCrae, 1989). Finally, unstructured written feedback from community participants in IMPROVment classes suggested that participants showed greater body awareness of self and others, as well as less judgment of self and others after regular participation in class. Therefore, we included the Philadelphia Mindfulness Scale (PHLMS) (Cardaciotto et al., 2008), a mindfulness measure that assesses attentional awareness and acceptance.

### MRI Measures

#### MRI Acquisition

All participants received 30-min MRI scans at baseline and follow-up. MRI scans were acquired on a research-dedicated 3.0T Siemens Skyra scanner with a 32-channel headcoil (Siemens, Germany). High resolution T1-weighted images were acquired using an MPRAGE-GRAPPA sequence (TR = 1,900 ms, TE = 2.93 ms, TI = 900 ms, flip angle = 9 degrees, 176 slices). Resting state blood-oxygen level dependent (BOLD) functional magnetic resonance imaging (fMRI) images were acquired using a whole-brain gradient-echo echo planar imaging sequence (35 contiguous slices; slice thickness = 3.75 mm; in-plane resolution = 3.75 × 3.75 mm, TR = 2.0).

#### fMRI Preprocessing

Images were preprocessed using an established pipeline (Neyland et al., 2021). Segmentation of the T1-weighted structural image into gray matter, white matter and cerebrospinal fluid was done using Statistical Parametric Mapping version 12 (SPM12, http://www.fil.ion.ucl.ac.uk/spm). The resulting images were used to create a whole-brain mask for using during warping. The mask was manually edited using MRIcron software (https://www.nitrc.org/projects/mricron) to ensure that all extra-brain tissue was excluded prior to warping. Editing of the mask was performed by one trained study staff member and confirmed by a second. All images were warped to the Montreal Neurological Institute (MNI) template using Advanced Normalization Tools (ANTs, https://www.nitrc.org/projects/ants).

The first 10 volumes of the BOLD image were removed to eliminate irregularities in signal due to the stabilization of the magnetic field. Distortion correction was done in FMRIB's Software Library (FSL, www.fmrib.ox.ac.uk/fsl) to address BOLD effect induced inhomogeneities in the magnetic field that can cause signal loss and SPM12 was used for slice timing correction and realignment. Potential confounding for non-neuronal signal due to motion and other physiological signals was addressed by applying motion scrubbing, a bandpass filter (0.009–0.08 Hz), and adjusting for average signal from the gray matter, white matter, and cerebrospinal fluid as well as movement parameters. BOLD images were coregistered to their native space structural image and then warped to the MNI template using the same parameters used to warp the T1 structural image to MNI space.

#### Brain Network Generation

To study functional connections and communication within the brain, a network, or graph, was created for each individual from the fMRI data (Telesford et al., 2011). Connectivity was measured as the Pearson's correlation coefficient between nodes *i* and *j* using the fMRI time series for each node pair. A whole-brain functional connectivity matrix was generated by performing the correlation for all node pairs. This connectivity matrix was then binarized into an adjacency matrix (*Aij*) by only keeping positive edges which ensure a predefined edge density (S = log (N)/log (K)) can be maintained across participants. The majority of analyses used a threshold of S = 2.5 based on both our data here and previous work showing that this threshold produces networks with minimal fragmentation Clauset et al., 2004 and density ratios which most closely compare to other naturally generated networks Sporns, 2018. We also assessed network fragmentation across multiple thresholds of S (1.5, 2.0, 2.5, and 3.0) with an increase in S resulting in a sparser adjacency matrix and greater potential for fragmentation (Clauset et al., 2004). The giant component, the largest group of nodes which can be connected by a single path, was used to assess fragmentation. All analyses of efficiency were calculated on the full binarized adjacency matrix and modularity analyses were performed on the giant component using S = 2.5.

Briefly, several key functional network variables were calculated (for review see Sporns, 2018). Global efficiency (*E*_Glob_) represents how efficiently information from one node can move through the whole network (Latora and Marchiori, 2001). Local efficiency (*E*_Loc_) reflects how efficiently information from one node can move to its neighbors (Latora and Marchiori, 2001). Communities or modules refer to regions of the brain that are more connected to each other than other nodes within the network. The network was partitioned into communities, with each voxel assigned to a single community, by optimizing the overall modularity of the brain calculated as *Q* (Girvan and Newman, 2002). Consistency of community structure was measured within regions of interest (ROI) using Scaled Inclusivity (SI) (Steen et al., 2011). SI values range from 0 to 1 where high SI values indicate that a particular network community is consistently observed within the group (Moussa et al., 2012). Regional changes in whole brain metrics and community analyses were done on two *a priori* ROIs, the somatomotor cortex (SMC) and default mode network (DMN). The SMC is involved in motor planning and execution and the DMN has been implicated in mood disorders (Brakowski et al., 2017) and AD (Buckner et al., 2009). ROIs were generated using resting-state brain networks from independent data in 22 normal young adults from a prior study (Mayhugh et al., 2016).

### Statistical Analyses

This study was undertaken to assess feasibility and effect sizes of potential hypotheses. Given the small sample size, statistical analysis was limited to descriptive statistics and simple models. *P*-values are reported in **Tables 2**–**4** and should be interpreted with caution. All statistical analysis were evaluated using the *stats* package in R 4.1.1 (Team, 2021) using RStudio (Team, 2020). Means and standard deviations of demographic information are presented in Table 1. A two tailed *t*-test was used to demonstrate the lack of statistically significant differences in scores at baseline between the groups (Table 2). Paired *t*-tests were used to evaluate outcome measures before and after intervention (Table 2).

**Table 1:** Demographic information for PWD and CG enrolled in the pilot.

	**Caregivers**			**People with Dementia**		
	**Control (*n* = 5)**	**Intervention (*n* = 5)**		**Control (*n* = 5)**	**Intervention (*n* = 5)**	
	**Mean (SD)**	**Mean (SD)**	***p*-value**	**Mean (SD)**	**Mean (SD)**	***p*-value**
Age	74.77 (5.20)	72.50 (5.58)	0.523	79.63 (4.79)	74.15 (8.28)	0.236
Female	4 (80%)	2 (40%)	0.24	1 (20%)	4 (80%)	0.125
Married	5 (100%)	5 (100%)		5 (100%)	5 (100%)	
Education	15 (3.32)	17.80 (1.48)	0.123	14.60 (2.41)	18 (2)	0.041*
MCI/AD (*n*)	NA	NA		3/2	3/2	

*Results reported either as mean (SD) or n (%)*.

*The asterisk was used to represent a p-values below 0.05 from the statistical analyses*.

**Table 2:** Mean values at baseline and follow-up for non-imaging and imaging outcomes in PWD.

	**Control (*****n*** **= 5)**			**Intervention (*****n*** **= 5)**			
	**Baseline**	**Follow-up**		**Baseline**	**Follow-up**		
	**Mean (SD)**	**Mean (SD)**	***p*-value**	**Mean (SD)**	**Mean (SD)**	***p*-value**	**Baseline comparison (*p*-value)**
**Non-imaging outcomes**							
FAB	26.4 (7.67)	21.8 (5.89)	0.12	28.8 (6.76)	30 (5.43)	0.43	0.61
FES	23.8 (3.42)	27.2 (9.09)	0.27	24 (6.59)	27.4 (9.04)	0.27	0.95
GDS	2.2 (1.48)	2.2 (1.92)	0.07	1.2 (1.64)	1.8 (1.48)	1	0.34
NPI_D+A_	4 (4.90)	5 (3.87)	0.44	5.5 (6.19)	6.75 (3.77)	0.63	0.85
PHLMS-Awareness	23.6 (5.2)	20.6 (6.5)	0.02*	23.2 (7.2)	28.4 (7.9)	0.004*	
PHLMS-Acceptance	24.2 (2.5)	24.0 (4.9)	0.94	23.2 (5.9)	21.4 (4.7)	0.22	
QoL-AD	42.2 (5.02)	41.4 (6.23)	0.47	42.4 (1.52)	43 (1.58)	0.55	0.93
**Whole-brain imaging outcomes**							
Giant component size	18,500 (1,795)	18,070 (1,567)	0.26	18,254 (2,041)	19,569 (1,224)	0.26	0.84
Degree	53.79 (0.04)	53.80 (0.05)	0.79	53.82 (0.04)	53.79 (0.04)	0.29	0.34
E_glob_	0.18 (0.04)	0.17 (0.04)	0.30	0.17 (0.05)	0.21 (0.03)	0.27	0.81
E_loc_	0.48 (0.04)	0.47 (0.03)	0.79	0.46 (0.04)	0.50 (0.04)	0.18	0.58
Path length	5.79 (1.55)	6.05 (1.21)	0.42	6.27 (2.22)	4.92 (0.97)	0.28	0.70
Clustering	0.31 (0.01)	0.32 (0.02)	0.13	0.31 (0.02)	0.32 (0.02)	0.33	0.63
Modularity (Q)	0.69 (0.05)	0.69 (0.05)	0.97	0.63 (0.04)	0.69 (0.04)	0.13	0.06
**SMC regional outcomes**							
Path length	56.84 (35.38)	63.17 (28.86)	0.38	60.75 (32.79)	37.30 (24.44)	0.26	0.86
E_glob_	0.14 (0.04)	0.13 (0.03)	0.39	0.13 (0.04)	0.16 (0.03)	0.30	0.75
E_loc_	0.36 (0.07)	0.41 (0.03)	0.85	0.34 (0.04)	0.38 (0.05)	0.29	0.57
SI	0.02 (0.01)	0.02 (0.01)	0.16	0.02 (0.01)	0.04 (0.02)	0.05*	0.80
**DMN regional outcomes**							
Path length	47.23 (24.56)	53.40 (22.50)	0.36	49.96 (30.21)	29.21 (15.20)	0.22	0.88
E_glob_	0.15 (0.03)	0.14 (0.03)	0.43	0.15 (0.04)	0.18 (0.02)	0.24	0.89
E_loc_	0.40 (0.02)	0.36 (0.04)	0.83	0.40 (0.04)	0.44 (0.01)	0.15	0.87
SI	0.04 (0.03)	0.05 (0.02)	0.53	0.04 (0.01)	0.05 (0.02)	0.40	0.74

*The asterisk was used to represent a p-values below 0.05 from the statistical analyses*.

**Figure 1:**
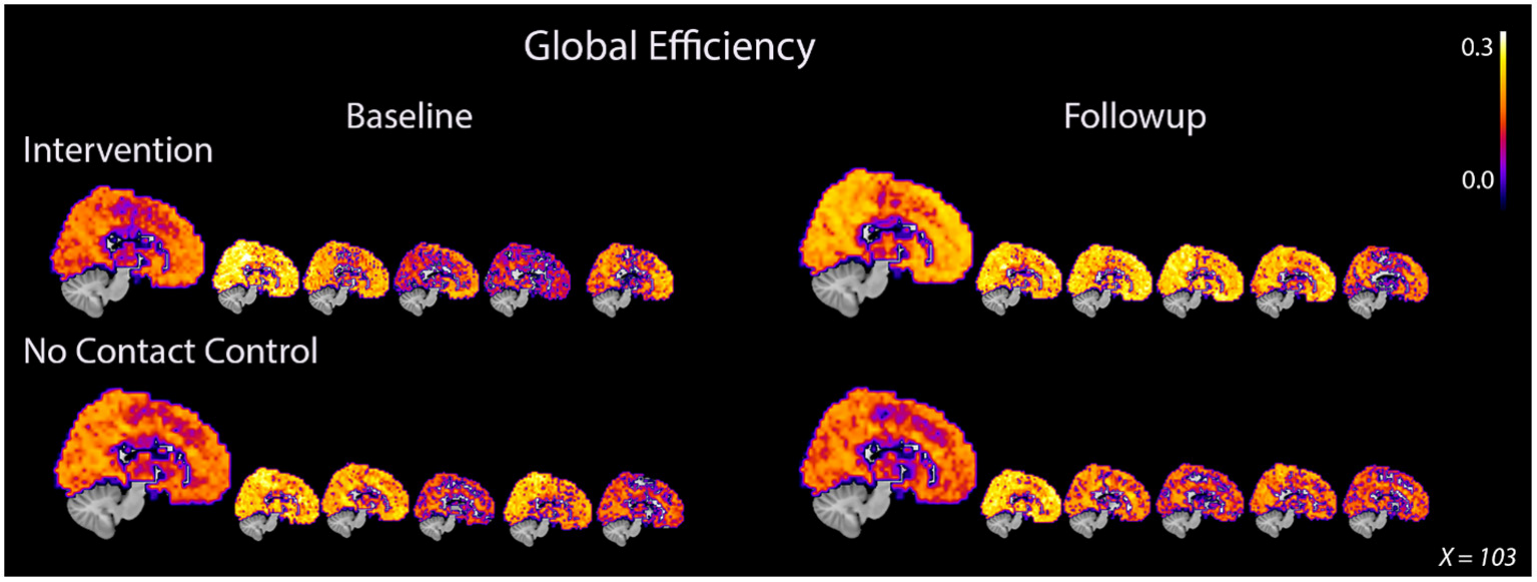
Global efficiency consistently increased in PWD after 8 weeks of dance intervention. PWD who participated in the intervention demonstrated an average increase in global efficiency after intervention while the control participants showed an average decrease. The increase in whole brain efficiency was demonstrated by all five participants in the intervention group. Participants in the control group showed either no decrease or a decrease in global efficiency after 8 weeks. The large image represents an average image for the group while each of the smaller images represents an individual participant. The same midsagittal slice is used for each image.

Between-group differences assessed using a simple linear regression analysis are presented for completeness but should be interpreted with caution due to the small sample size (Table 3). For voxel-wise imaging analysis, the main objective was to observe if changes in connectivity were consistent across different metrics and across participants. This is conveyed visually in Figure 1 where group averages are shown followed by individual results. Pearson's correlation tests were used to evaluate how changes in neural networks are related to changes in non-imaging metrics (Table 4).

**Table 3:** Regression analysis results estimating the effect of intervention on imaging and non-imaging metrics in PWD.

	**Beta values**	**95% CI**	***p*-values**
**Balance outcomes**			
FAB	6.46	1.11, 11.81	<0.05*
FES	−0.07	−9.05, 8.91	>0.20
**Mood**			
GDS	0.46	−1.04, 1.96	>0.20
NPI (Depression + Apathy)	0.09	−6.17, 6.36	>0.20
NPI(D+A) adjusted for CG Neuroticism	2.07	−7.68, 3.51	>0.20
**Quality of life**			
QoL-AD	1.38	−2.26, 5.04	>0.20
**Network metrics**			
Giant component size	1,590	−297, 3,476	0.08
Degree	−0.008	−0.08, 0.05	>0.20
Global efficiency	0.04	−0.01, 0.09	0.10
Local efficiency	0.03	−0.02, 0.08	0.16
Path length	−1.24	−2.84, 0.35	0.11
Clustering	0.002	−0.02, 0.02	>0.20
Modularity (Q)	0.01	0.05, 0.08	>0.20
**Scaled inclusivity**			
DMN	0.001	−0.03, 0.03	>0.20
SMC	0.02	−0.005, 0.04	0.12
**Global efficiency**			
DMN	0.03	0.003, 0.07	0.08
SMC	0.03	−0.02, 0.08	0.16
**Local efficiency**			
DMN	0.03	−0.004, 0.07	0.08
SMC	0.03	−0.05, 0.10	>0.20

*The asterisk was used to represent a p-values below 0.05 from the statistical analyses*.

**Table 4:** Correlations between changes in non-imaging and imaging metrics in PWD.

	**Correlation value**	***p*-value**
**QoL**		
QoL-AD and FAB	0.27	>0.20
QoL-AD and FES	−0.41	0.20
QoL-AD and GDS	−0.60	0.07
QoL-AD and NPI_D+A_	−0.26	>0.20
**Balance and the somatomotor network**		
FAB and SI	0.46	0.17
FAB and Eloc	−0.07	>0.20
FAB and Eglob	0.01	>0.20
**Mood and the default mode network**		
NPID+A and SI	0.90	0.10
NPID+A and Eloc	0.01	0.50
NPID+A and Eglob	−0.59	>0.20

## Results

### Participant Demographics

Of the 10 dyads enrolled, nine were spousal couples and one was sisters. One dyad in the intervention group identified as Black and the remainder identified as White. Age ranged from 64 to 85 years and overall, CG were slightly younger (age = 73.6 ± 5.2) than MCI/PWD (76.9 ± 7.0). Slightly over half of the participants were women (6 CG, 5 MCI/PWD). Overall, the groups were well-matched; however, the intervention group tended to have higher educational attainment than the control group (Table 1). Each group contained two participants adjudicated as having early-stage AD and three participants adjudicated as having MCI. The overall attendance rate for the pilot study was 96.25% due to two dyads absence from the first class and an additional absence from a third dyad.

#### Participant Feedback

Anonymous qualitative feedback was solicited from participants who completed the intervention. Responses to the question “Has this course directly increased your life satisfaction or happiness?” included that the class “loosens me up,” “made me laugh and have fun,” and “energy, patience, listening skills.” Responses about the favorite parts of class included “interactions with other couples,” “new ways to move, making new friends,” and “Our teacher's support, creativity, seeing others improvise, compassion for others.”

### Non-imaging Metrics

The outcomes of the study are reported below and in Tables 2, 3. *P*-values are included in the tables for completeness, but are not meaningful due to the small sample size and any interpretation should be done with caution.

#### Balance

FAB scores increased by 1.2 points, from 28.8 ± 6.76 to 30 ± 5.43, in the intervention group and decreased by 4.6 points, from 26.4 ± 7.67 to 21.8 ± 5.89, in MCI/PWD in the control group (Table 2, Supplementary Figure S1). A one point increase in FAB score is estimated to decrease fall risk by ~8% in older adults (Hernandez and Rose, 2008). Therefore, the intervention reduced fall risk by an estimated 9.6% while fall risk increased by ~36.8% in the control groups. This results in an average difference of 5.8 points or ~46.4% risk between the two groups. Both the control group and the dance intervention group demonstrated an increased score on the FES that did not differ between groups (Table 2). A regression analysis showed that between-group improvements in balance, as measured by the FAB, were statistically significant (Table 3).

#### Mood

Average GDS score at follow-up increased slightly, from 1.2 ± 1.64 to 1.8 ± 1.48, in the intervention group and showed no change in the control group (Table 2, Supplementary Figure S1). Average follow-up NPI_D+A_ scores increased slightly (5.5 ± 6.19–6.75 ± 3.77) in the intervention group and slightly more (4.0 ± 4.90–5 ± 3.87) in the control group (Table 2, Supplementary Figure S1). A planned regression model was run on the NPI_D+A_ score adjusting for the neuroticism score on the NEO-FFI. CG neuroticism changed the valence and effect size of NPI score, suggesting it is important to consider in the full trial (Table 3). None of these differences reached statistical significance (Table 3). Furthermore, changes in these two measures of mood were not well-correlated (Table 4).

#### Quality of Life

QoL-AD scores increased in the dance intervention, from 42.4 ± 1.53 to 43 ± 1.58, and decreased in the control group, from 42.2 ± 5.02 to 41.4± 6.23 (Table 2, Supplementary Figure S1). Regression analysis showed a positive effect of intervention on QoL that did not reach statistical significance (Table 3). Correlations were calculated between change in QoL-AD and change in FAB, FES, GDS, and NPI_D+A_ across all 10 MCI/PWD subjects to provide information on whether and how changes in balance and mood correlated with changes in QoL (Table 4). A modest correlation (r = 0.27) was observed between change in QoL and changes in balance measured with the FAB, with a slightly stronger correlation observed with changes in balance confidence measured on the FES (r = −0.41) (Table 4). Better QoL was also correlated with improved NPI depression and apathy (r = −0.26) and with decreased GDS scores (r = −0.60) (Table 4).

### Imaging Metrics

#### Whole Brain Network Metrics

On average, *E*_Loc_ increased slightly after intervention, 0.46 ± 0.04–0.50 ± 0.04, and decreased slightly in the control group, from 0.18 ± 0,04 to 0.17 ± 0.04 (Table 2, Supplementary Figure S2). Whole-brain *E*_Glob_ also improved after intervention, from 0.17 ± 0.05 to 0.21 ± 0.03 (Table 2, Supplementary Figure S2). Average and per-individual spatial maps of *E*_Glob_ before and after intervention are shown in Figure 1. Regression analyses on both whole-brain local and global efficiency are shown in Table 3. A change in Q value does not provide information about the spatial patterns of the communities, but previous work suggests Q values may increase in response to exercise intervention (Baniqued et al., 2017). We observed increased Q values in the intervention group, 0.63 ± 0.04–0.69 ± 0.04, that were two orders of magnitude greater than the average increase in Q observed in the control participants, from 0.69 ± 0.05 to 0.69 ± 0.05 (Table 2, Supplementary Figure S2). Similarly, Q did not change significantly in response to the intervention (Table 3).

#### Regional Network Analyses

**Figure 2:**
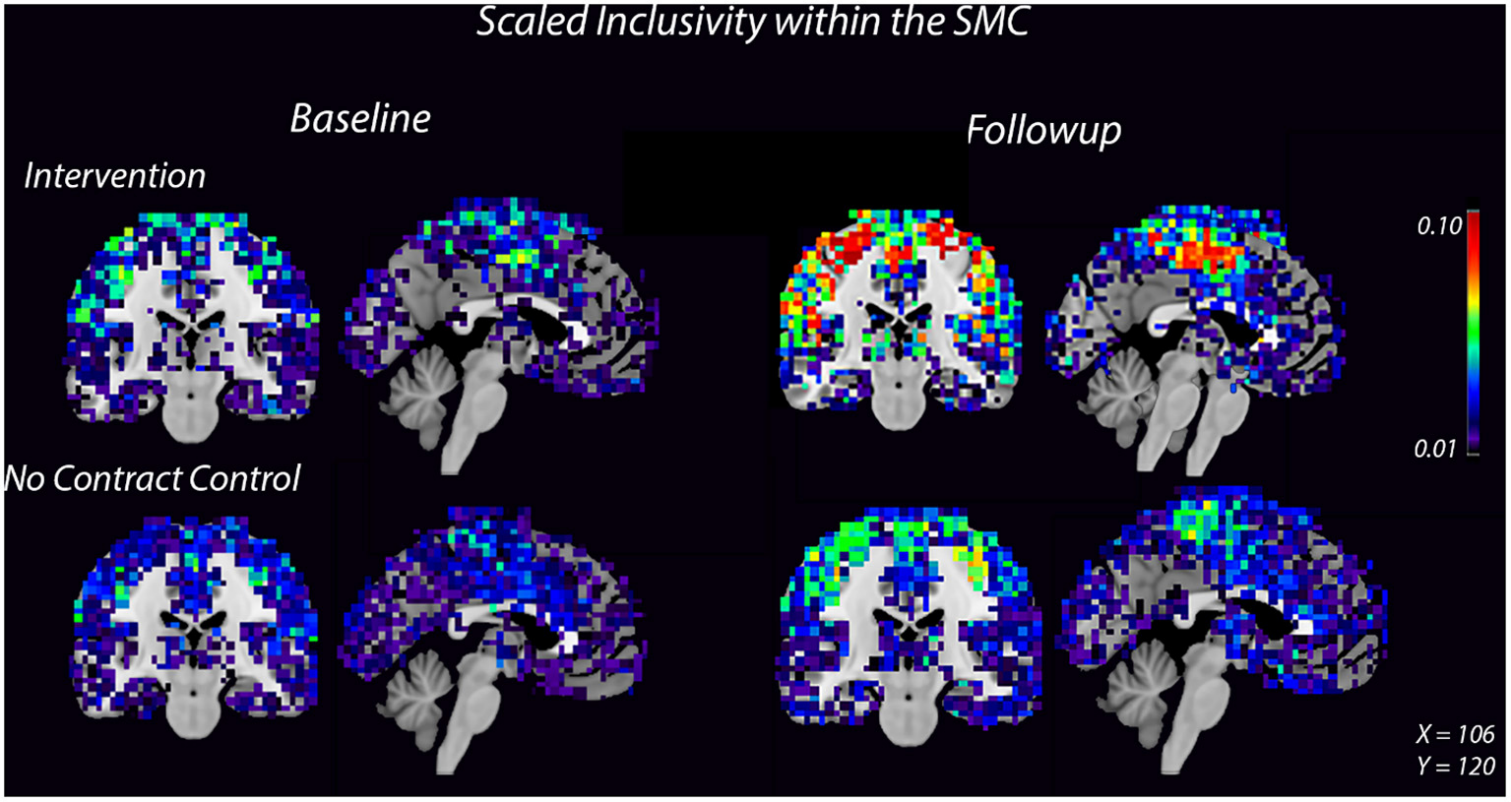
Consistency of the SMC community increases after 8 weeks of dance intervention. Warm colors show higher consistency. Consistency within the SMC increased in the intervention group compared to the control group. For each group, the left image is a coronal slice which shows bilateral motor cortices and the right image is a midsagittal slice which shows medial SMC.

High spatial consistency of modules is observed in healthy young adults (Hugenschmidt et al., 2014). SI within the SMC increased in the intervention group, from 0.02 ± 0.01 to 0.04 ± 0.02, with no increase observed in the control group (Figure 2, Supplementary Figure S2). There was a small SI increase in the DMN that did not differ between the control and intervention groups (Table 2, Supplementary Figure S2). *E*_Glob_ increased 25% in the SMC and 20% in the DMN in the intervention group, compared with slight decreases in both regions in the control group. In the SMC, *E*_Loc_ increased slightly in both groups (Table 2), while in the DMN, *E*_Loc_ increased in the intervention group, from 0.40 ± 0.04 to 0.44 ± 0.01, and decreased in controls, from 0.40 ± 0.02 to 0.36 ± 0.04, (Table 2). This change in local efficiency within the DMN after intervention trends toward significance (Table 3).

#### Associations Between Local Brain Networks and Balance, Mood, and QoL

##### Balance

Over all 10 MCI/PWD participants, change in FAB was fairly well-correlated with changes in SMN SI (r = 0.46) but was not strongly correlated with either changes in global or local efficiency within the SMN (Table 4). Correlations between changes in FES and SMN network measures were also relatively weak (r <0.20) (Table 4).

##### Mood

Correlations between change in GDS and changes in network measures in the DMN scores across all 10 participants were relatively weak (r < 0.30) (Table 4). Across both intervention groups, change in NPI_D+A_ was not significantly correlated with change in network measures (Table 4). However, in MCI/PWD who completed the intervention, changes on NPI_D+A_ were strongly correlated with changes in DMN SI (r = 0.90) as well as E_glob_ (r = −0.59) (Table 4).

### Network Structure

#### Giant Component Size

**Figure 3:**
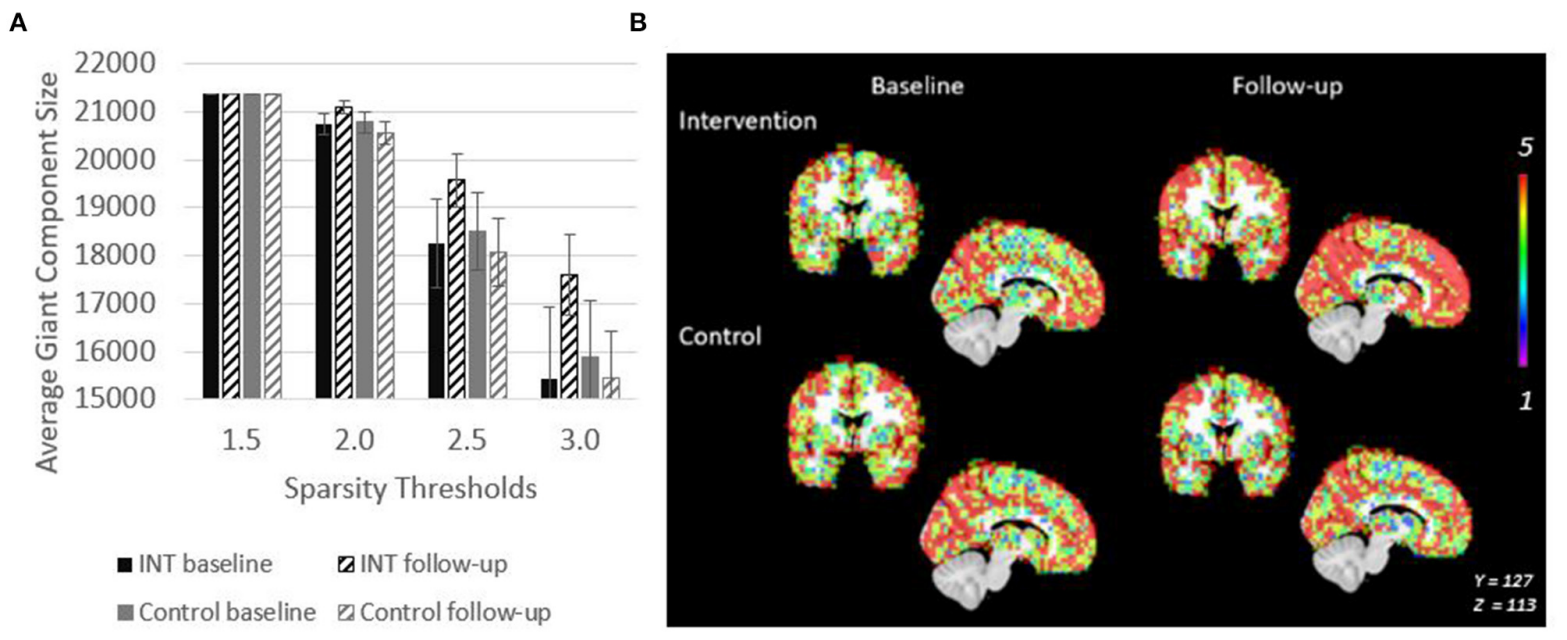
Giant Component size increases after intervention in PWD. **(A)** Giant component size across sparsity thresholds are shown to demonstrate that the intervention related effect on size is observable across a range of network thresholds. **(B)** Spatial overlaps of the giant component across all five participants in each group are shown. Red represents voxels in which every participant had a node in that region. Purple represents voxels in which only one participant had a node in that region.

The giant component at S = 2.5, a commonly used sparsity threshold for brain imaging networks (Hayasaka and Laurienti, 2010), increased in size after intervention in MCI/PWD an average of 7% as compared to the 2% decrease in size observed in the control group (Table 2). A linear regression analysis at S = 2.5 shows that the effect of the intervention on giant component size trends toward significance in MCI/PWD (*p* = 0.08) (Table 3). The size of the giant component is directly related to the sparsity threshold; a lower sparsity threshold generates a denser network with a larger giant component. Therefore, we investigated whether changing the sparsity threshold altered the trend for increased giant component size after intervention. Figure 3A shows giant component size before and after intervention at four sparsity thresholds. Except the most liberal threshold, the average giant component size in the intervention group increased after the intervention and decreased slightly in the control group, with larger differences noted at higher thresholds (Figure 3A).

Spatial maps of the giant component were created at a sparsity threshold of S = 2.5 to explore the spatial distribution of added nodes (Figure 3B). The value in the voxels in this image show the number of individuals within the group (maximum of *n* = 5) who have a node of the giant component at that location. After intervention, nodes were not added in any clear pattern, but seemed to be distributed across the brain. This resulted in an overall more consistent spatial distribution across the participants as evidenced by the increased number of voxels with a value of 5 (Figure 3B). This effect is not seen in the control group.

## Discussion

The pilot study demonstrated feasibility of a twice-weekly improvisational movement intervention. Ten dyads (20 participants) were recruited within 4 weeks. The five dyads who completed the intervention had excellent attendance and positive qualitative feedback about their experience, including specific feedback about social connection as a benefit of participation. This supports work by Marchant and colleagues that reported positive feedback and good attendance for improvisational dance classes in older adults (Marchant et al., 2010). There were no adverse events related to the intervention during the study.

The results reported here are from a pilot study with a very small sample size. They were meant to inform design choices, not to establish efficacy. The discussion below focuses on the descriptive changes that were observed and relates them to the existing literature. It cannot be assumed that the outcomes observed here will be replicated in a larger sample. Dance has been shown to improve balance and mobility in older people with Parkinson's disease (e.g., Hackney and Earhart, 2010; Batson et al., 2014) or dementia (Koh et al., 2020) as well as healthy older adults (Eyigor et al., 2009; Kattenstroth et al., 2010). Consistent with these findings, participation in the improvisational dance intervention resulted in improved scores on balance which resulted in a 9.6% reduced risk of falling in MCI/PWD while participants in the control group demonstrated a 33% increased risk of falling. The decrease in balance in the control group was larger than expected. To the best of our knowledge, there were no differences in data collection or participant injuries outside of the intervention that could explain the change.

Previous dance interventions have demonstrated a betterment in mood in older adults (Eyigor et al., 2009). Here, self-reported scores on the GDS did not show an improvement in either group. CG-reported symptoms of apathy and depression worsened slightly in both groups, but with a smaller increase in the dance intervention group. The effect size of this relationship increased substantially after adjusting for CG neuroticism, a personality trait. This result should not be interpreted as a meaningful finding, but it does suggest that accounting for CG personality when relying on CG-reported outcomes may be an important consideration for a larger trial. Overall, the pilot results suggest that the dance intervention may have the potential to improve quality of life and mood by providing an enjoyable, social, movement-centered environment for MCI/PWD.

Preliminary findings reported here support the idea that positive changes can occur over 8 weeks in brain networks of individuals with an active neurodegenerative disease. Graph theory analysis of brain networks showed increased efficiency and consistency in brain network architecture in MCI/PWD who completed the intervention as compared to control participants. Global efficiency increased across the brain, as well as locally in the SMC and DMN, regions of interest. These findings are interesting given that individuals with AD generally experience a decrease in global and local efficiency (Tijms et al., 2013; Dennis and Thompson, 2014). Similarly, modular organization of the brain also increased in the intervention group. Again, modular organization has been shown to reduce with age and pathology (Contreras et al., 2019) and to increase in response to exercise (Baniqued et al., 2017). Eight weeks of dance increased Q values two orders of magnitude as compared to the average change observed in the control group. In addition, the consistency of the SMC community increased after the dance intervention. Increased community consistency within this region has been shown to occur after exercise in older adults and is tied to positive outcomes (Petrie et al., 2017).

Balance increased after the intervention and change in balance was correlated with changes in community consistency in the SMC. However, change in FAB was not well-correlated with changes in global or local efficiency. Community consistency within the DMN correlated strongly with depression and apathy on the NPI, but no network measures in the DMN correlated well with quality of life. These findings are interesting given recent results suggesting that DMN connectivity may be linked to social engagement in people with depression (Saris et al., 2020). Together, these findings highlight the need for further exploration into the relationship between regional network metrics and both mobility and mood in MCI/PWD. The full trial will explore these relationships with a larger sample and additional measures.

An unexpected observation was that the size of the giant component, the largest collection of interconnected nodes within the brain network, increased after the dance intervention. All participants passed basic fMRI quality control for head motion and artifacts, were preprocessed to adjust for non-neural signal using accepted methods and had a giant component within the expected size range at baseline and follow-up at the standard sparsity threshold of S = 2.5. To assess whether changes in giant component size were related to the sparsity threshold, multiple thresholds were evaluated. At three of the four thresholds, the giant component increased after intervention. More sparse networks had a larger increase in giant component size. The spatial layout of the giant component showed that more nodes within the SMC were included in the giant component after intervention. The reason for the increase in giant component size is not clear. It is possible that the giant component increased due to shifts in correlations in neural activity. For example, given that the same threshold was used before and after intervention, the additional nodes could have increased relative connection strength to survive the threshold after the intervention. It is also possible that changes in blood flow or vascular regulation could have changed in response to the intervention. Given that the BOLD signal used in fMRI uses blood oxygenation as a proxy for neural activity, a brain-wide vascular shift could be reflected in the correlations used to construct the networks. While increased cerebral perfusion has been observed in response to aerobic exercise (Burdette et al., 2010; Stillman et al., 2021), these changes are usually small, likely because the cerebral blood flow is tightly regulated to protect against changes in oxygenation (Querido and Sheel, 2007). Further work and replication is needed to confirm and disambiguate this observation.

## Conclusions

This pilot study was used to assess feasibility and effect sizes to inform design of a clinical trial investigating how dance affects mood, balance, and brain networks in MCI/PWD. Due to the limited sample size in this pilot, statistically significant between-group differences were not expected. However, the pilot provided valuable insight into both imaging and non-imaging metrics which can be further investigated in future studies.

## Data Availability Statement

The raw data supporting the conclusions of this article will be made available by the authors, without undue reservation.

## Ethics Statement

The studies involving human participants were reviewed and approved by Wake Forest School of Medicine Institutional Review Board. The patients/participants provided their written informed consent to participate in this study.

## Author Contributions

CS and CH contributed to the conception and design of the study. PB and RK made intellectual contributions to implementation of data collection and quality control. CH wrote the first draft of the manuscript. DT, CS, and RB wrote the sections of the manuscript. DT, RL, and CH analyzed pilot data. PL and EI guided analysis and contributed to interpretation of pilot data and methods. All authors contributed to manuscript revision, read, and approved the submitted version.

## Funding

This publication was made possible by funding through BlueCross BlueShield North Carolina through a pilot grant to support wellness and from the Wake Forest University Translational Science Center. Support for REDCap Software and the Clinical Research Unit were provided through the Wake Forest CTSI UL1TR001420.

## Author Disclaimer

The contents of this publication are solely the responsibility of the authors and do not necessarily represent the official views of any funder.

## Conflict of Interest

The authors declare that the research was conducted in the absence of any commercial or financial relationships that could be construed as a potential conflict of interest.

## Publisher's Note

All claims expressed in this article are solely those of the authors and do not necessarily represent those of their affiliated organizations, or those of the publisher, the editors and the reviewers. Any product that may be evaluated in this article, or claim that may be made by its manufacturer, is not guaranteed or endorsed by the publisher.
