# Supplementary material for: Improvisational Movement to Improve Quality of Life in Older Adults With Early-Stage Dementia: A Pilot Study
Source: Front Sports Act Living. 2022 Jan 14;3:796101. doi: 10.3389/fspor.2021.796101 (PMC8795741; doi:10.3389/fspor.2021.796101)
Supplement: Supplementary file 1 [file Data_Sheet_1.docx]

**Supplementary Figure 1. Nonimaging metrics at baseline and follow-up for all PWD**

**
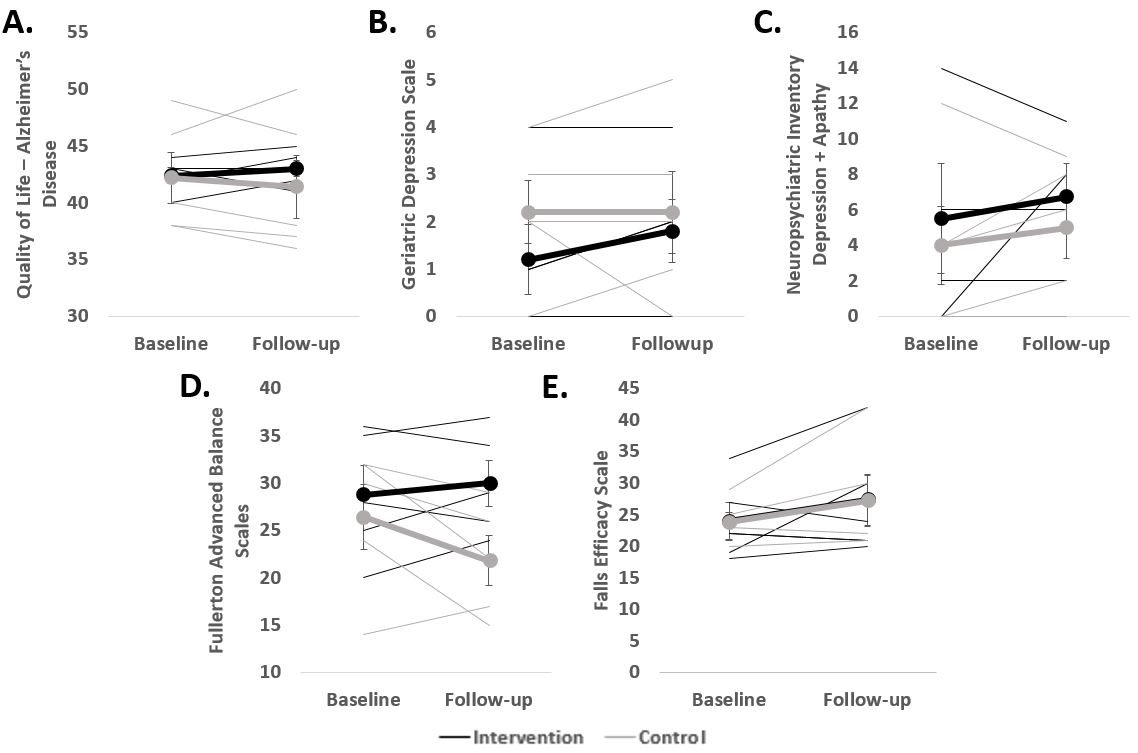
**

Light weight lines show the scores on nonimaging outcome metrics at baseline and followup for all participants. Heavier weighted lines indicate average scores at the two time points with error bars representing standard error. Lines in black represent intervention participants whereas lines in gray represent control participants.

**Supplementary Figure 2. Imaging metrics at baseline and follow-up for PWD**

**
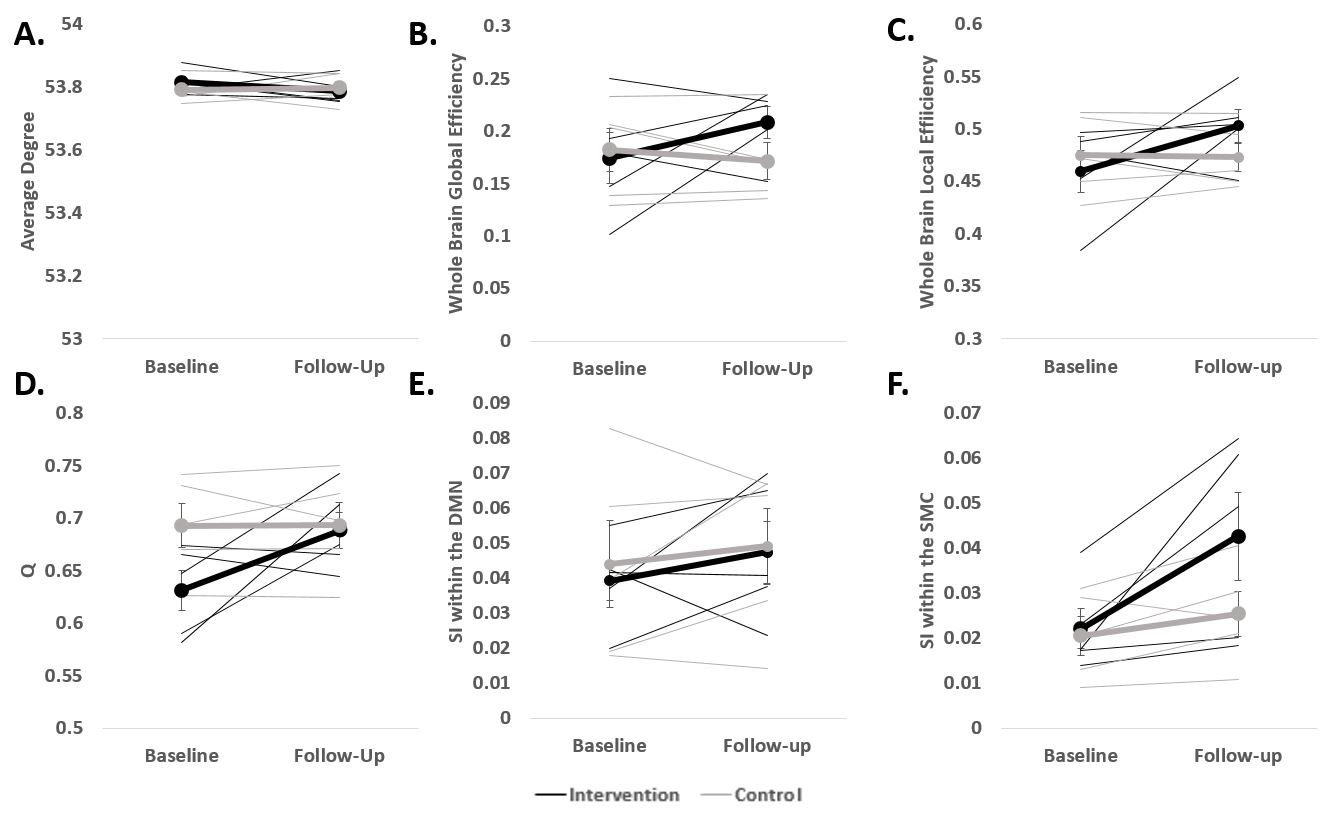
**

Light weight lines show the scores on imaging outcome metrics at baseline and followup for all participants. Heavier weighted lines indicate average scores at the two time points with error bars representing standard error. Lines in black represent intervention participants whereas lines in gray represent control participants.
